# ROBOTIC ASSISTED VERSUS LAPAROSCOPIC DISTAL PANCREATECTOMY: A RETROSPECTIVE STUDY

**DOI:** 10.1590/0102-672020230065e1783

**Published:** 2023-12-08

**Authors:** Ricardo JUREIDINI, Guilherme Naccache NAMUR, Thiago Costa RIBEIRO, Telesforo BACCHELLA, Lucas STOLZEMBURG, José JUKEMURA, Ulysses RIBEIRO, Ivan CECCONELLO

**Affiliations:** 1 Universidade de São Paulo, São Paulo State Cancer Institute, Department of Gastroenterology – São Paulo (SP), Brazil.; 2 Univesidade de São Paulo, Faculty of Medicine, Department of Gastroenterology – São Paulo (SP), Brazil.

**Keywords:** Pancreatectomy, Robotic Surgical Procedures, Costs and Cost Analysis, Laparoscopy, Morbidity, Pancreatectomia, Procedimentos Cirúrgicos Robóticos, Custos e Análise de Custo, Laparoscopia, Morbidade

## Abstract

**BACKGROUND:**

Minimally invasive distal pancreatectomy (MIDP) is associated with less blood loss and faster functional recovery. However, the benefits of robotic assisted distal pancreatectomy (RDP) over laparoscopic distal pancreatectomy (LDP) are unknown.

**AIMS:**

To compare RDP versus LDP for surgical treatment of benign lesions, pre-malignant and borderline malignant pancreatic neoplasias.

**METHODS:**

This is a retrospective study comparing LDP with RDP. Main outcomes were overall morbidity and overall costs. Secondary outcomes were pancreatic fistula (PF), infectious complications, readmission, operative time (OT) and length of hospital stay (LOS).

**RESULTS:**

Thirty patients submitted to LDP and 29 submitted to RDP were included in the study. There was no difference regarding preoperative characteristics. There was no difference regarding overall complications (RDP – 72,4% versus LDP – 80%, p=0,49). Costs were superior for patients submitted to RDP (RDP=US$ 6,688 versus LDP=US$ 6,149, p=0,02), mostly due to higher costs of surgical materials (RDP=US$ 2,364 versus LDP=1,421, p=0,00005). Twenty-one patients submitted to RDP and 24 to LDP developed pancreatic fistula (PF), but only 4 RDP and 7 LDP experienced infectious complications associated with PF. OT (RDP=224 min. versus LDP=213 min., p=0.36) was similar, as well as conversion to open procedure (1 RDP and 2 LDP).

**CONCLUSIONS:**

The postoperative morbidity of robotic distal pancreatectomy is comparable to laparoscopic distal pancreatectomy. However, the costs of robotic distal pancreatectomy are slightly higher.

## RESUMO

**RACIONAL:**

A pancreatectomia distal minimamente invasiva (PDMI) está associada a menos perda sanguínea e recuperação funcional mais rápida, no entanto, os benefícios da pancreatectomia distal robótica (PDR) são desconhecidos quando comparada a pancreatectomia distal laparoscópica (PDL).

**OBJETIVOS:**

Comparar PDR versus PDL no tratamento cirúrgico de lesões benignas, neoplasias pancreáticas malignas, pré-malignas e limítrofes.

**MÉTODOS:**

Estudo retrospectivo comparando PDL com PDR. Os desfechos primários avaliados foram morbidade e custos hospitalares. Os desfechos secundários foram fístula pancreática (FP), complicações infecciosas, readmissão, tempo cirúrgico e tempo de internação hospitalar (TIH).

**RESULTADOS:**

Trinta pacientes submetidos a PDL e 29 submetidos a PDR foram incluídos no estudo. Não houve diferença em relação às características pré-operatórias. Não houve diferença em relação às complicações gerais (PDL – 72,4% versus PRD – 80%, p=0,49). Os custos foram superiores para PDR (PDR=US$ 6688 versus PDL=US$ 6149, p=0,02), principalmente devido aos custos mais elevados de materiais cirúrgicos (PDR=US$ 2364 versus PDL=1421, p=0,00005). Vinte e um pacientes submetidos a PDR e 24 submetidos a PDL desenvolveram fístula pancreática (PF), no entanto, apenas 4 submetidos PDR e 7 a PDL apresentaram complicações infecciosas associadas a FP. O tempo cirúrgico (PDR=224 min. versus PDL=213 min., p=0,36) e a conversão para cirurgia aberta (1 PDR e 2 PDL) não tiveram diferença estatística.

**CONCLUSÕES:**

A morbidade pós operatória da pancreatectomia distal robótica é comparável à pancreatectomia distal laparoscópica. Entretando, os custos da pancreatectomia distal robótica são mais elevados.

## INTRODUCTION

The first laparoscopic pancreatoduodenectomy was reported in 1994^17^. Two years later, the first laparoscopic distal pancreatectomy (LDP) was performed for chronic pancreatitis^10^. Recently, two well designed randomized clinical trials^6,33^ comparing minimally invasive distal pancreatectomy (MIDP) with open distal pancreatectomy (ODP) confirmed that MIDP is related with less blood loss, shorter length of hospital stay and shorter time to functional recovery; however, the very few robotic distal pancreatectomies (RDP) were included in the multicentre LEOPARD trial^33^.

Although MIDP has been proved safe and even superior to ODP regarding short-term outcomes for benign or borderline malignant pancreatic lesions^2,9^, less then 40% of all distal pancreatectomies performed in the USA are minimally invasive^32^. This could be attributed to a relatively long and tough learning curve for LDP^8^. Robotic-assisted surgery may aid surgeons to overcome a few limitations related LDP, because the commercially available robot-assisted surgical platforms offer three-dimensional vision, better ergonomics and tremor-free surgical instruments that provide a broad range of movements, even greater than the human hand^4^.

The first robotic-assisted distal pancreatectomy (RDP) was reported by Melvin in 2003^26^. Since then, RDP has been a matter of interest for several authors who demonstrated that RDP is feasible and safe. However, there is some degree of uncertainty regarding advantages over LDP, and major concern about costs related to the introduction of this somewhat new technology^14,21,31,35^.

The objective of the present study was to compare short-term surgical outcomes and cost of LDP and RDP for patients with benign, pre-malignant and borderline malignant pancreatic neoplasia.

## METHODS

### Study design

This is a retrospective single-institution study, conducted at a tertiary hospital in Brazil, comparing short-term surgical outcomes of LDP and RDP for treatment of benign, pre-malignant and borderline malignant pancreatic neoplasia. The study was approved by the Ethics Committee of the Institution (Certificate of Presentation for Ethical Appreciation — CAAE 21934413.2.0000.0065).

### Patients

From February 2015 to February 2020, consecutive patients scheduled for minimally invasive distal pancreatectomy for treating benign, pre-malignant and borderline malignant pancreatic neoplasia were selected from a prospective maintained database. Patients’ assignment either to LDP or RDP depended on the availability of the Da Vinci^®^ Surgical System.

### Inclusion criteria

The study included patients aged between 18 and 80 years old, with American Society Anesthesiology (ASA) score equal or less than III, with benign, pre-malignant or borderline malignant neoplasia located in the body or tail of the pancreas.

### Exclusion criteria

Patients with lesions involving the celiac axis, common hepatic artery or porto-mesenteric confluence were not considered for MIDP. Although abutment or incasement of splenic vessel was not a contraindication by itself, patients with severe collateral circulation after splenic venous thrombosis were not scheduled for MIDP. Pregnancy, breastfeeding or mental illness which could preclude patients from signing surgical consent were also considered exclusion criteria.

### Surgical procedure

All RDP and LDP were performed by two experienced pancreatic surgeons, each one performing more than 30 pancreatic resections every year, and both having overcome the learning curve for MIDP before the start of the study. Both were certified by Intuitive as console surgeons, and although they had already performed other robotic-assisted procedures, a certified proctor tutored them through the first three RDP.

**Figure 1:**
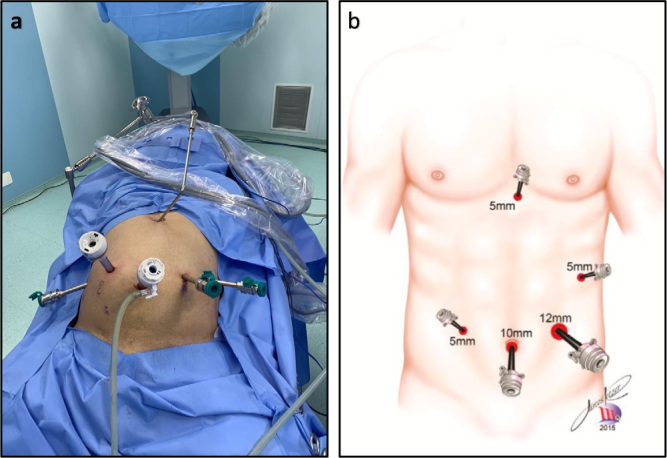
Trocars placement: (a) Trocar placement for robotic distal pancreatectomy; (b) Trocar placement for laparoscopic distal pancreatectomy.

For LDP, pneumoperitoneum was induced using a Verres needle whenever possible. Trocars were disposed according to Figure 1b. The surgeon was located between the patient’s abducted legs. Afterwards, gastrocolic ligament and the short gastric vessels were divided with an advanced bipolar energy device, and the stomach and liver were retracted upwards using a Nathanson Liver Retractor^®^. Next, splenic artery was ligated with Hem-o-lok^®^ clips. A “window” between the splenic vein and pancreas was dissected to transect the pancreatic parenchyma. Wherever considered feasible by the surgeon, a laparoscopic surgical stapler was applied (Echelon FlexTM, blue cartridge, Johnson & Johnson). The stapler’s jaw was closed very slowly to avoid any tear of pancreatic parenchyma. When a stapler was not used, the pancreatic stump was closed with a “U” shape interrupted suture. Afterwards, splenic vein was also ligated with Hem-o-lok^®^ clips, the splenic colonic flexure was mobilized, and the distal pancreas and spleen were detached from the retroperitoneum.

For RDP, trocar disposal is shown in Figure 1a. A midline 12 mm trocar was used for the Da Vinci^®^ Si Scope. A Da Vinci^®^ Si Surgical System was used in all RDP and a patient-side cart was always docked over the patients’ head. After docking, division of the gastrocolic ligament and short gastric vessels was done with a harmonic scalpel, but the rest of dissection was performed with a permanent cautery hook. Hem-o-lok clips were applied by the bedside assistant. Transection of the pancreatic parenchyma was performed in the same way as in LDP.

Spleen preservation was conducted at the surgeons’ discretion only for benign lesions, such as serous cystic neoplasia or small insulinomas. For borderline malignant or pre-malignant neoplasia spleen preservations was not attempted. The surgical specimen was removed from the abdominal cavity with a disposable bag through a Pfannenstiel incision. An abdominal drain was placed near the pancreatic stump in all patients.

### Postoperative care

Diet was resumed on postoperative day one. Patients were discharged from hospital if pain was mild and manageable with oral medications, if patient tolerated solid diet and if laboratory exams showed no sign of infection. Drains were only removed when amylase levels were inferior to three times the upper limit of serum amylase, not before postoperative day seven.

### Endpoint

The main endpoins of this study were overall postoperative complications and overall hospital costs. Complications were defined as any deviation from the normal postoperative course and were classified according to Clavien-Dindo^12^. Total hospital cost was divided into seven different categories; surgical postoperative unit cost, intensive care unit cost, operating room length of stay, personnel, exams performed during postoperative internment like computed tomography scan, costs with materials like staplers, disposable advanced energy devices, robotic instruments and the robotic plastic cover, and also medication costs. Costs were expressed in US dollars. Expenses with acquisitions of laparoscopic and robotic system were excluded from the analyses.

Secondary endpoints were severe complications (Clavien-Dindo ≥3), pancreatic fistula (PF), defined according to the 2016 International Study Group of Pancreatic Fistula (ISGPS) classification as a drain output of rich amylase fluid, three times the upper limit of serum amylase, causing mild deviation in posteoperative course (type B), or severe complications, as organs failure or reoperation (type C)^3^. Other secondary endpoints analysed were fistula-related infectious complications, conversion to open procedure, operative time, blood transfusion and length of stay, reoperation and readmission.

### Statistical analysis

Continuous variables were expressed as means and were compared using a t-test or Mann-Whitney test. Qualitative variables were expressed as frequencies and compared with a c2 test. A p=0,05 was considered significant and the Statistical Package for the Social Sciences (SPSS) program (SPSS in, Chicago, IL) was used for the analysis.

## RESULTS

Sixty consecutive patients were assigned for MIDP for treatment of benign, pre-malignant and borderline malignant pancreatic neoplasia, 30 for RDP and 30 for LDP. One patient in the RDP group that had a supposed pancreatic cyst was excluded from the analysis because, during initial exploration, an extra-pancreatic lesion was found.

Demographic and clinical details are summarized in Table 1. Age and sex distribution did not differ between both groups. Mean body-mass index (BMI) was 27,3 kg/m^2^ for RDP and 29,4 kg/m^2^ for LDP, and this difference was not statistically significant. Preoperative diagnosis and ASA classification was also not different in both groups.

**Table 1:** Demographic and clinical preoperative data.

	RDP (n=29)	LDP (n=30)	p
Age in years (SD)	47.6±14	49.8±14	0.55
Sex, n (%)			
Female	25 (86.2)	24 (80)	0.73
Male	4 (13.8)	6 (20)	
BMI kg/m^2^ (SD)	27.3±4.73	29.4±6.43	0.15
Preoperative diagnosis, n (%)			
Cystic neoplasia	16 (55.2)	14 (46.6)	0.77
PNET	11 (37.9)	15 (50)	
Other	2 (6.9)	1 (3.4)	
ASA classification, n (%)			
1	5 (17.2)	7 (23.3)	0.35
2	22 (75.9)	18 (60)	
3	2 (6.9)	5 (16.7)	
Diabetes	6 (20.7)	4 (13.3)	0.50
Smoking	7 (24.1)	4 (13.3)	0.28

RDP: robotic distal pancreatectomy; LDP: laparoscopic distal pancreatectomy; SD: standard deviation; BMI: body mass index; PNET: pancreatic neuroendocrine tumor; ASA: American Society of Anesthesiologist classification.

Table 2 summarizes intraoperative data and short-term outcomes. There were three conversions to open procedure, one in the RDP group and two in the LDP group, all of them caused by intensive inflammatory process which prevented progress. Only one LDP needed blood transfusion throughout the study. Usage of staplers and operative time did not differ between groups.

**Table 2:** Intraoperative details and short-term outcomes.

	RDP (n=29)	LDP (n=30)	p
Operative time (minutes)	224±54	213±49	0.36
Conversion to open procedure n (%)	1 (3.4)	2 (6.7)	1.00
Blood transfusion n (%)	0	1 (3.4)	1.00
Spleen preservation n (%)	0	2 (6.7)	0.492
Parenchyma transection with stapler n (%)	24 (82.8)	23 (76.7)	0.56
Overall morbidity n (%)	21 (72.4)	24 (80)	0.49
Clavien-Dindo n (%)			
1	17 (58.6)	16 (53.3)	0.091
2	3 (10.3)	7 (23.3)	
3	1 (3.4)	1 (3.3)	
Pancreatic fistula	21 (72.4)	24 (80)	0.493
B	20 (69)	23 (76.7)	
C	1 (3.4)	1 (3.3)	
Fistula/Infectious complications	4 (13.8)	7 (23.2)	0.384
Reoperations, n (%)	1 (3.4)	1 (3.3)	1
Readmission, n (%)	3 (10.3)	4 (13.3)	1
Lenght of stay in day	6.6±1.6	7.2±3.4	0.94
Diagnosis, n (%)			
PNET	10 (34,5)	18 (60)	
SCN	2 (6.9)	1 (3.3)	
MCN	8 (27.6)	4 (13.3)	
IPMN	6 (20.7)	4 (13.3)	
Other*	3 (10.3)	3 (10)	
Tumor size (cm)	3.58±2.4	3.77±2.51	0.94
Lymph nodes resected	9.8±7.8	11.3±6.8	0.338

RDP: robotic distal pancreatectomy; LDP: laparoscopic distal pancreatectomy; PNET: pancreatic neuroendocrine tumor; SCN: Serous Cystic Neoplasia; MCN: Mucinous Cystic Neoplasia; IPMN: Intraductal P apillary Mucinous Neoplasm; *benign cyst, solitary fibrous tumor, desmoid tumor, solid pseudopapillary pancreatic tumor.

Overall morbidity was not different in both groups (Table 2), 72.4% of RDP and 80% of LDP had at least one complication. Only one patient in each group had severe complications (Clavien Dindo=3). One patient in each group needed reoperation due to infected abdominal collection not manageable through interventionist radiology. PF was the single most relevant complication, occurring in 21 (72.4%) of the RDP patients and 24 (80%) of the LDP. However, most patients only required drainage for a period greater than three weeks, and only four (13.8%) of RDP and seven (23.2%) of LDP experienced infectious complications related to PF. One LDP with a grade C PF also had a pulmonary embolism and another LDP had a reversible acute renal injury. There was no mortality or any other complication in both groups. Length of stay (6.6 days for DRP versus 7.2 days for LDP) and readmissions (10.3% for DRP versus 13.3% for LDP) were also not different between groups. Pathologic findings are summarized in Table 2.

Overall costs were superior for RDP (Table 3), US$ 6,688 versus US$ 6,149 for LDP (p=0.02). Surgical materials, which included the robotic surgical instruments, were the main responsible for this lower than US$ 600 difference. The amount spent with disposable materials was US$ 2,364 for RDP and US$ 1,421 for LDP (p=0.00005).

**Table 3:** Costs of both procedures.

	RDP (n=29)	SD	LDP (n=30)	SD	p
SPOCU costs, US$	1,288.95	288.8	1,287.31	582.92	0.178
ICU costs US$	210.92	338.96	309.69	381.47	0.518
Operating room length of stay cost, US$	594.12	131.8	907.34	176,03	0.0001
Personnel, US$	1,774.21	324.22	1,711.12	572,76	0.139
Exams, US$	178.56	130.93	253.28	241,60	0.181
Materials, US$	2,364.12	807.1	1,421.7	440,59	0.00005
Medications, US$	275.55	80.5	259.02	470,93	0.0002
Total costs, US$	6,688.97	1,236.22	6149.49	1,862,68	0.020

RDP: robotic distal pancreatectomy; SD: standard deviation; LDP: laparoscopic distal pancreatectomy; SPOCU: surgical postoperative unity; ICU: intensive care unit; US$: American dollars.

## DISCUSSION

This study demonstrated that short-term surgical outcomes for LDP and RDP were equivalent, for benign pre-malignant and borderline malignant pancreatic neoplasia, but costs were slightly superior for RDP mainly because of expenses with surgical materials. Although this was not a randomized trial, there was no difference between groups when comparing demographics and clinical data.

In a recent meta-analysis, Kamarajah et al. concluded that RDP is related to longer operative time, probably associated with docking and undocking procedures^20^. Even though mean docking time in the RDP group was 6 min (data not shown), overall operative time was not superior to LDP and was comparable with others previous reports from different institutions^7,13,15^. Therefore, this shows that operating the robotic surgical platform was not a serious burden for surgical staff. Although RDP has been associated with lower conversion rate than LDP to open procedure in previous studies^28^, the conversion rate was similarly low in both groups.

Operative time is usually considered an important outcome while evaluating the learning curve of a procedure^27^. The number of cases to overcome the learning curve for minimally invasive distal pancreatectomy is variable among different studies^5,8,27^. In 2022, Müller et al., in a systematic review, concluded that the number of procedures needed to be proficient was 16 cases for LDP and 15 for RDP. However, previous surgical experience by surgeons and institutions in minimally invasive surgery, especially in those studies evaluating robotic surgery, was unknown, and this may represent a major bias^22^. When this study was started, all surgeons had already overcome the learning curve for LDP.

The parenchyma transection method was chosen by surgeon discretion. However, in most procedures a laparoscopic surgical stapler was applied, because it is not related to greater occurrence of PF^11^. Overall complications were not different between groups and PF was the major driver for morbidity in this study. Although PF occurred in most cases in both groups, this was largely due to a very conservative policy of abdominal drain management, and after the update of the ISGPS consensus for PF, all patients with drainage longer than three weeks should be assigned as grade B PF, despite the absence of symptoms or alterations in clinical course^3^. Only 19% of patients in RDP and 30% in LDP experienced infectious complications related to PF and one patient in each group demanded reoperations for uncontrolled abdominal collections, although none of these patients presented any organic dysfunction. The severity of complications was slightly higher in LDP; however, this was not statistically relevant, and the prevalence of grade 3 or higher complications was equivalent to that reported by other authors^13,15,16,24,30^. Readmission is not an unusual event during the postoperative course of distal pancreatectomies^34^. Three patients in the RDP and four in the LDP group were readmitted for treatment of surgical site infection related to PF. PF was the only responsible for the high complications rate and the majority of patients had the diagnosis of this disease solely because an abdominal drain was kept in place for longer than three weeks. However, hard outcomes like sepsis, reoperation or readmission were equally rare in both groups, and were compatible with previous studies^1,7,13,15,16,25^.

Total hospital costs were higher for RDP, mainly due to greater expenses with surgical materials, although the magnitude of such difference was rather smaller than expected. Robotic surgery is usually associated with greater expenses, especially because of costs like the patient’s cart cover or the semi-disposable Endowrist^®^ instruments^16,29,30^; however, the price of acquisition and maintenance of the robotic surgical platform is never considered because it is diluted by intensive usage in high volume centers. Although RDP was not superior to LDP in this study and costs were marginally higher, there may be some benefits of using robotic surgery for distal pancreatectomy. First, although there is robust evidence in favor of minimally invasive distal pancreatectomy, less then 60% of these procedures are performed in such way^23^; therefore, this technology may assist introducing minimally invasive surgery to unfamiliar professionals, as it is related to shorter learning curves and may enhance surgical skills due to 3rd dimensional cameras and surgical graspers with full range of motion, greater than the human hand^27^. Second, RDP may be an introduction to more complex minimally invasive pancreatic surgery, such as central pancreatectomies or Whipple procedures. In a recent randomized controlled trial, laparoscopic Whipple procedure was associated with greater morbidity and mortality, and maybe robotic surgery could help overcome the difficulties related to the execution of such complex tasks, especially with the development of new technologies such as new advanced bipolar devices, augmented reality and image-guided pancreatic surgery^18,19^.

This study had many limitations mainly because it was retrospective, therefore not randomized, which may have caused several biases, although preoperative data was not different between the RDP and LDP groups. Moreover, as it is a single-institutional study, results may not be applicable elsewhere. Finally, the acquisition cost of the robotic system and laparoscopic systems was not included in the analysis; however, it is very difficult to estimate how much those equipments would cost per patient, as the overall price is diluted by progressive usage.

## CONCLUSIONS

The overall complications of RDP are not different from LDP, and costs are slightly higher mainly because of greater expenses with disposable materials.
